# Unilateral Sensation Loss of the NAC After Superomedial Pedicle Reduction Mammaplasty

**Published:** 2015-07

**Authors:** Elif Sari

**Affiliations:** Kirikkale University Faculty of Medicine, Department of Plastic, Reconstructive and Aesthetic Surgery, Kirikkale, Turkey

**Keywords:** Nipple-areola complex, Sensation, Reduction mammaplasty

**DEAR EDITOR**

Superomedial pedicle reduction mammaplasty has become the most preferred technique in our clinic. While performing the technique, the surgeon can predict the final breast volume and shape, as well as limit sensation loss of the nipple-areola complex (NAC).^1^ Another advantage of this procedure is the improved long-term projection of the breast. Moreover, the technique obtains younger-looking breasts with elevated inframammary folds.^1^

A total of 40 patients were operated by using superomedial pedicle reduction mammaplasty technique last year in our clinic. However, sensation loss of the NAC has not been reported except one patient. The aim of this letter is to report a unilateral sensation loss of the NAC of a patient who underwent reduction mammaplasty by using superomedial pedicle technique.

A 33-year-old female patient without any systemic disease was admitted to our clinic with macromasty (Figure 1a). Her body mass index was 27 kg/m^2^. The distance between the sternal notch-to-nipple was 30 cm in right breast, and 31 cm in left one before the surgery. The classical superomedial pedicle reduction mammaplasty was performed. A total of 310 g soft tissue was removed in right breast, and 315 g in left one. The NAC transposition was 10 cm in right breast and 11 cm in left breast. No complication was occured during or after the surgery such as hematoma, infection, seroma, wound healing problem or NAC necrosis. 

**Fig. 1:**
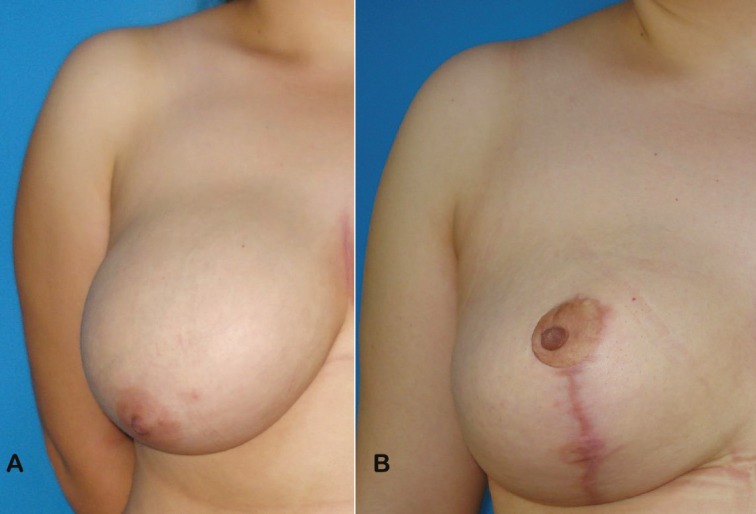
Picture of a 33-year-old female patient who underwent superomedial pedicle reduction mammaplasty. **(a)** Preoperative frontal view of the right breast, and **(b)** postoperative 6th month frontal view of the right breast were demonstrated in these figures

The patient was followed-up for 12 months. In the first month visit, she suffered from sensation difference between two NAC of the breasts. Complete sensation loss of the right NAC was detected in Semmes-Weinstein monoflaments test. The sensation loss was not resolved during the other visits in 3rd, 6th and 12th months (Figure 1b). This complication could not based on any reason such as nerve injury or traction of the soft tissues. Because, it was reported that the NAC is only innervated by the third, fourth, or fifth intercostal nerves.^2^

Reduction mammaplasty is an effective surgical procedure to improve the quality of life, especially in severe gigantomasty patients.^3^ However, loss of sensation of the NAC is a major challange for plastic surgeons. Heine *et al.*^4^ treated 25 gigantomasty patients by using Lejour technique, and reported a reduced NAC sensation in 8 patients (32%). However, Nahabedian *et al.*^5^ reported a 98% retained NAC sensation after medial-pedicle reduction mammaplasty.

Similarly, Lugo *et al.*^1^ reported 98% normal sensation of the NAC in 200 patients treated with superomedial pedicle reduction mammaplasty. To the best of our knowledge, unilateral sensation loss of the NAC in superiomedial reduction mammaplasty was not reported before. Actually, we could not understand the reason of this complication and decided to share this case with our colleagues.

## CONFLICT OF INTEREST

The authors declare no conflict of interest.

## Note

Please cite this paper as:

Sari E. Unilateral Sensation Loss of the NAC After Superomedial Pedicle Reduction Mammaplasty. World J Plast Surg 2015;4(2):175-176.

## References

[1] Lugo LM, Prada M, Kohanzadeh S, Mesa JM, Long JN, Torre J (2013). Surgical outcomes of gigantomastia breast reduction superomedial pedicle technique. Ann Plast Surg.

[2] Kuzbari R, Schlenz I (2007). Reduction mammaplasty and sensitivity of the nipple-areola complex Sensuality versus sexuality?. Ann Plast Surg.

[3] Spear ME, Nanney LB, Phillips S, Donahue R, Rogers KM, Wendel JJ, Summit B, Kelly K, Shack RB, Hagan KF (2012). The impact of reduction mammaplasty on breast sensation. Ann Plast Surg.

[4] Heine N, Eisenmann-Klein M, Prantl L (2008). Gigantomasty: treatment with a short vertical scar. Aesthetic Plast Surg.

[5] Nahabedian MY, McGibbon BM, Manson PN (2000). Medial pedicle reduction mammaplasty for severe mammary hypertrophy. Plast Reconstr Surg.

